# Preoperative endothelial dysfunction in cutaneous microcirculation is associated with postoperative organ injury after cardiac surgery using extracorporeal circulation: a prospective cohort study

**DOI:** 10.1186/s13613-020-00789-y

**Published:** 2021-01-07

**Authors:** Stanislas Abrard, Olivier Fouquet, Jérémie Riou, Emmanuel Rineau, Pierre Abraham, Cyril Sargentini, Yannick Bigou, Christophe Baufreton, Sigismond Lasocki, Samir Henni

**Affiliations:** 1 Department of Anesthesiology and Intensive Care, University Hospital of Angers, Angers, France; 2 Vascular Medicine, University Hospital of Angers, Angers, France; 3 MITOVASC Institut, INSERM 1083 - CNRS 6015, University of Angers, Angers, France; 4 Department of Anesthesiology and Critical Care Medicine, Edouard Herriot Hospital, Hospices Civils de Lyon, Lyon, France; 5 Department of Cardiac Surgery, University Hospital of Angers, Angers, France; 6 Micro Et Nanomedecines Translationnelles, MINT, UMR INSERM 1066, UMR CNRS 6021, University of Angers, Angers, France; 7 Sports Medicine, University Hospital of Angers, Angers, France; 8 Département d’Anesthésie Réanimation, Hôpital Édouard Herriot, Hospices Civils de Lyon, 5 place d’Arsonval, 69437 Lyon CEDEX 03, France

**Keywords:** Endothelial function, Preoperative evaluation, Cardiac surgery, Organ dysfunction

## Abstract

**Background:**

Cardiac surgery is known to induce acute endothelial dysfunction, which may be central to the pathophysiology of postoperative complications. Preoperative endothelial dysfunction could also be implicated in the pathophysiology of postoperative complications after cardiac surgery. However, the relationship between preoperative endothelial function and postoperative outcomes remains unknown. The primary objective was to describe the relationship between a preoperative microcirculatory dysfunction identified by iontophoresis of acetylcholine (ACh), and postoperative organ injury in patients scheduled for cardiac surgery using cardiopulmonary bypass (CPB).

**Methods:**

Sixty patients undergoing elective cardiac surgery using CPB were included in the analysis of a prospective, observational, single-center cohort study conducted from January to April 2019. Preoperative microcirculation was assessed with reactivity tests on the forearm (iontophoresis of ACh and nitroprusside). Skin blood flow was measured by laser speckle contrast imaging. Postoperative organ injury, the primary outcome, was defined as a Sequential Organ Failure Assessment score (SOFA) 48 h after surgery greater than 3.

**Results:**

Organ injury at 48 h occurred in 29 cases (48.3%). Patients with postoperative organ injury (SOFA score > 3 at 48 h) had a longer time to reach the peak of preoperative iontophoresis of acetylcholine (133 s [104–156] vs 98 s [76–139] than patients without, *P* = 0.016), whereas endothelium-independent vasodilation to nitroprusside was similar in both groups. Beyond the proposed threshold of 105 s for time to reach the peak of preoperative endothelium-dependent vasodilation, three times more patients presented organ dysfunction at 48 h (76% vs 24% below or equal 105 s). In multivariable model, the time to reach the peak during iontophoresis of acetylcholine was an independent predictor of postoperative organ injury (odds ratio = 4.81, 95% confidence interval [1.16–19.94]; *P* = 0.030).

**Conclusions:**

Patients who postoperatively developed organ injury (SOFA score > 3 at 48 h) had preoperatively a longer time to reach the peak of endothelium-dependent vasodilation.

*Trial registration* Clinical-Trials.gov, NCT03631797. Registered 15 August 2018, https://clinicaltrials.gov/ct2/show/NCT03631797

## Introduction

### Background

Microcirculation plays a central role in many essential life functions: coagulation, immunological functions such as diapedesis, inflammatory response, regulation of homeostasis, and vasomotor tone control. The capillary network is essential for the transport of nutrients and oxygen required for organ function.

Microcirculation alterations occur in several chronic diseases such as hypertension, diabetes mellitus and chronic renal disease [1, 2]. These diseases are associated with postoperative complications and organ dysfunction [3, 4]. The presence of preoperative microcirculatory alterations has also been associated with postoperative complications after non-cardiac surgeries. Gocke et al. found an association between preoperative endothelial function and cardiovascular events after vascular surgery [5, 6]. Additionally, cardiac surgery using cardiopulmonary bypass (CPB) is known to induce microcirculatory alterations. On one hand, cardiac surgery under CPB induces morphological alterations. Morphological modifications are characterized in sublingual video-microscopy by impaired capillary perfusion [7–9], glycocalyx thickness [7], perfusion heterogeneity and poorly perfused areas [10]. On the other hand, CPB induces microcirculatory functional alteration, also called endothelial dysfunction, as a decrease in endothelium-dependent and heat vasodilation [11]. These alterations are associated with elevated postoperative lactatemia independently of macrocirculatory parameters [8]. This side effect of CPB could amplify pre-existing microcirculatory alteration caused by chronic diseases.

The assessment of vascular functional responsiveness is not applicable by video-microscopy. Other non-invasive techniques, such as laser Doppler, are frequently used to measure microvascular blood flow. Among laser Doppler techniques, the laser speckle contrast imaging (LSCI) provides a two-dimensional perfusion index proportional to blood flow in relative units (mV) [12]. LSCI measurement of cutaneous skin blood flow is easily accessible and well correlated to others vascular beds [13, 14]. In order to assess their functional response capacity, capillaries should be stimulated to measure a change of blood flow. Iontophoresis is a method for non-invasive transdermal drug delivery based on the transfer of charged molecules using a low-intensity electric current. This method can be used to stimulate the capillaries. Combined with LSCI, acetylcholine (ACh) and sulfate nitroprusside (SNP) iontophoresis has been widely used to assess microvascular endothelial-dependent and -independent vasodilation, respectively [12, 15].

In cardiac surgery, preoperative measurements by video-microscopy are currently only used as baseline measurements for research. Furthermore, preoperative microcirculation measurements have never been used as a prognostic value for postoperative organ injury. Based on the simplicity of using LSCI and iontophoresis in a conscious patient during a consultation, we chose to use these techniques for preoperative microcirculatory assessment.

### Objective

The primary objective of this study was to describe the relationship between preoperative microcirculatory function, assessed by iontophoresis of acetylcholine and nitroprusside, and postoperative organ injury in a small patient population scheduled for cardiac surgery using CPB.

## Materials and methods

### Study design and ethics

The prospective observational cohort study MONS (microcirculation in cardiac surgery) was approved by institutional review board of Ile de France 1 (CPP IDF1; Hôtel-Dieu - 1 Place du Parvis Notre-Dame 75181 Paris CEDEX 04 France) (Chairperson Dr. C. Grillot-Courvalin) on 10 October 2018 (Ethical Committee No. 2018-A2341-54) and registered at Clinical-Trials.gov (NCT03631797) [16]. A signed informed consent was obtained from each patient. The study was reported according to the STROBE statement.

### Participants and setting

The cohort enrolled 60 patients scheduled for elective valvular or coronary cardiac surgery using CPB at our university hospital from January 2019 to April 2019. Patients with emergency surgery, combined surgery [valve and coronary arterial bypass grafting (CABG)], delirium or cognitive dysfunction before surgery were excluded from the MONS cohort. Patients with dark skin were also excluded because LSCI was not validated in this population yet.

### Variables

Patients’ preoperative characteristics including age, risk score in cardiac surgery EuroSCORE II [17], medical and surgical history, treatments and preoperative biological data were recorded. Intraoperative data including the use of vasoactive drugs, surgical time, CPB and aortic cross-clamp times were collected.

The primary endpoint was the occurrence of organ injury, defined as a Sequential Organ Failure Assessment (SOFA) score greater than 3 at 48 h after surgery. The SOFA score is used to track patients’ status during their stay in an intensive care unit (ICU) to determine the extent of organ failure [18, 19]. This score is composite and based on six different scores, one each for the respiratory, cardiovascular, hepatic, coagulation, renal and neurological systems. The choice of a cutoff at 3 in our study was supported by the association with poor outcomes after cardiac surgery. Furthermore, we chose to avoid the term of failure which relates to multi-organ failure associated with high mortality. The incidence of a multi-organ failure is too rare in cardiac surgery to base a preliminary study on it.

Secondary endpoints included the postoperative severity of illness SAPS II score [20] measured at 24 h after surgery, the evolution of SOFA at 24, 48 and 72 h, the peaks of arterial lactate and serum creatinine, the duration of use of catecholamines, and the length of ICU and hospital stay. The need for late surgical re-intervention (greater than 12 h) during the 30 postoperative days and the occurrence of atrial fibrillation or acute kidney injury ≥ stage 2 according to the KDIGO classification [21] during the hospital stay were recorded. The occurrence of other significant episodes including acute lung injury at 48 h after surgery (defined as a PaO_2_/FiO_2_ ratio less than 300 at 48 h) or hemodynamic failure (defined as the failure of catecholamines weaning within 48 h) were also recorded.

Safety and feasibility outcomes were monitored about the absence of adverse events or interference with clinical management; successful acquisition, signal processing and storage of skin perfusion data.

### Data sources and measurement

**Fig. 1:**
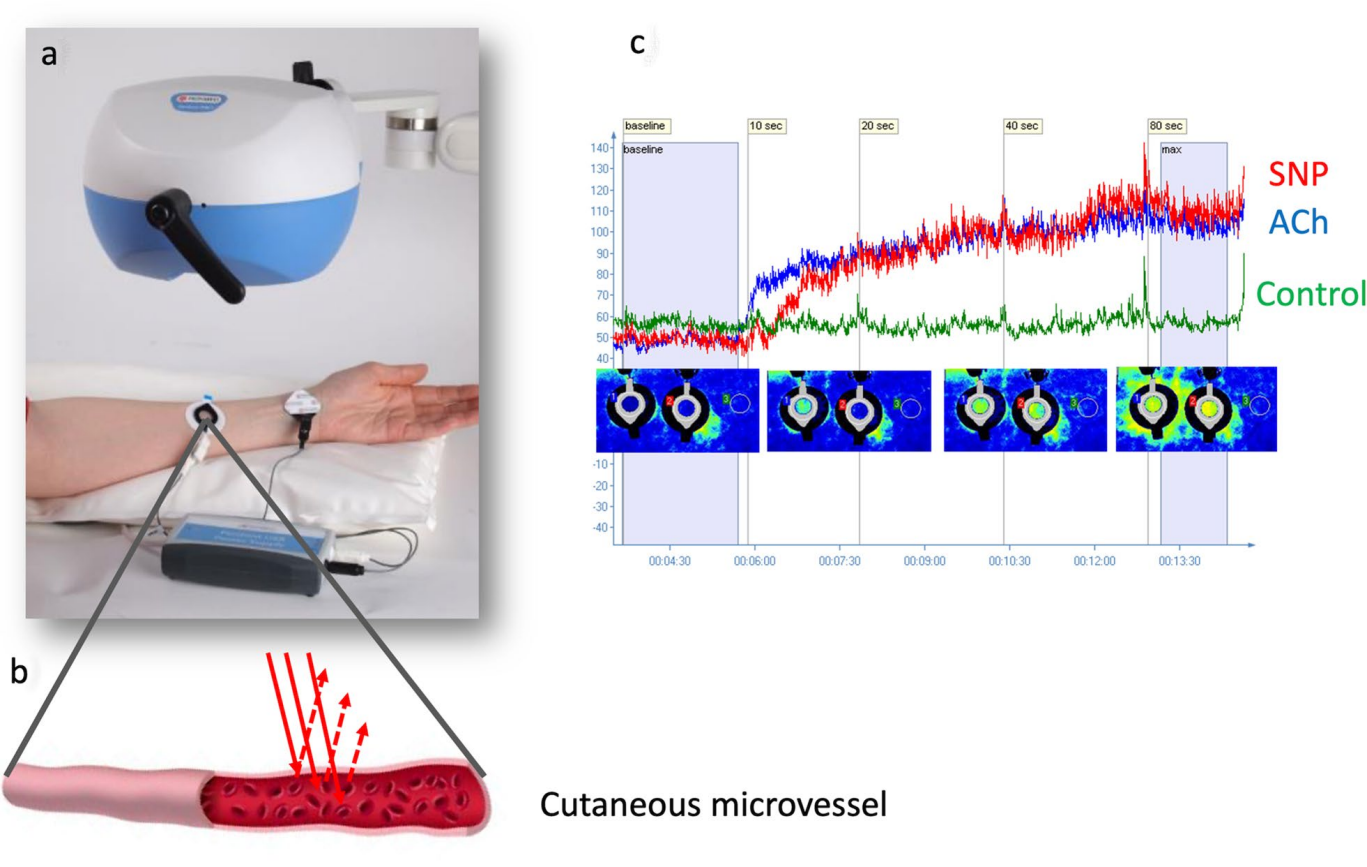
Evaluation of cutaneous microcirculation. **a** Iontophoresis in combination with blood perfusion imager. **b** Incident light from the laser (thick red arrows) are scattered back (dotted arrows) from red blood cells flowing in the microvessels. **c** Typical dose–response-related vasodilatation as a response to ACh (blue) and SNP (red)—blood perfusion imaging. Photos reproduced with the permission of Perimed (Järfälla, Sweden)

Microcirculation was evaluated for each patient a day before anesthesia and surgery. In a quiet consultation room at 22 ± 1 °C, a LSCI (Pericam PSI NR, Perimed, Järfälla, Sweden) was placed briefly 15 cm over the left forearm of patients, in the supine position, for non-invasive and continuous measurements of skin microvascular perfusion changes on 20-mm^2^ regions of interest (Fig. 1a). When laser light hit a matt surface, it generated a high-contrast, grainy pattern called the speckle pattern. Moving red blood cells make the speckle pattern fluctuate, and the contrast decreases as the velocity of the scatterers increases (Fig. 1b). LSCI could assess relative changes from a baseline in relative units (laser speckle perfusion units, LSPU = 10 mV) [12]. A Comfeel®/aluminum bilayer patch served as control [22]. Measurements were recorded with a frequency of 16 Hz and a resolution of 100 µm/pixel using an acquisition system (PIM Soft, Perimed, Järfälla, Sweden). After the measurement of baseline flow for 3 min, iontophoresis of ACh and SNP were used to stimulate skin microvessels in order to assess their endothelial-dependent and -independent reactivities, respectively [12, 15]. ACh 2% 200 μl and SNP 1% 200 μl (Sigma Aldrich, St. Louis, MO, USA) were delivered by the application of monopolar DC current of very low intensity (0.1 mA) using two small imbibed electrodes, anodal and cathodal, respectively, placed on the skin (electrodes LI 611, 3 M, Maplewood, USA). A PeriIont generator (Perimed, Järfälla, Sweden) was programmed to deliver a 2 mC current using an intensity of 0.10 mA with a duration of 20 s. Delivery automatically stopped at the end of the stimulation period. The microvascular response flow curves induced by ACh and SNP were recorded 5 min in order to reach the plateau response (Fig. 1c). Previous data indicated that the maximum responses occur during this time interval [23]. Recorded parameters were the peak amplitude (peak–baseline amplitude), the area under the curve (AUC) within 3 min of the microvascular flux and the time to reach the peak. Final filtered signal was obtained by subtracting movement artifact recorded on this region of interest [22].

### Bias

To reduce the interpatient variability, the patient course was standardized (see paragraph “Study procedure”). The results from the microcirculation evaluation were kept unknown to the surgical and intensive care team, and thus did not change patient management.

### Study procedure

Anesthesia was induced with intravenous propofol (target controlled infusion with a target plasma concentration of 2 to 3 µg/mL), sufentanil (target controlled infusion with a target cerebral site effect concentration of 1 ng/mL before sternotomy then 0.5 to 0.8 ng/mL after) and atracurium (bolus induction of 0.5 mg/kg repeated if necessary). Anesthesia was maintained by sufentanil, inhaled sevoflurane (MAC target of 0.8 to 1.0) during the non-bypass period instead of propofol during the CPB. In addition, bleeding was managed in the intraoperative period by the systematic use of a cell-saving device and an anti-fibrinolytic agent infusion (tranexamic acid intravenously administered over 60 to 120 min, 50 mg/kg or 100 mg/kg for patients with dual antiplatelet inhibition therapy). The mean arterial blood pressure during both CPB and non-CPB periods was maintained between 65 and 75 mmHg with norepinephrine infusion.

In a closed system (collapsible closed venous reservoir), heparin-coated CPB circuits (Carmeda, Medtronic, Minneapolis, MN, USA) or phosphorylcholine-coated CPB circuits (Phisio, LivaNova, Mirandola, Italy) with a membrane oxygenator and a cardiotomy reservoir were used. This circuit configuration nearly eliminates the blood–air interface for CABG surgery. Cardiotomy suction was used for transfusion of the mediastinal shed blood after treatment by the cell-saving device. CPB was performed under normothermia (> 36 °C) using a roller pump (Cobe, Lakewood, CO, USA) maintaining a flow rate of 2.4 L/min/m^2^. CPB priming (a mixture of Ringer’s solution and gelatin at 2:1 ratio) volume was reduced to a minimum using the retrograde autologous priming technique. Myocardial protection was kept constant throughout the period of investigation and was achieved by cold blood antegrade/retrograde cardioplegia (4:1 blood cardioplegia with Plegisol solution, Hospira, Paris, France). Once the patient was disconnected from CPB, the residual blood remaining in the extracorporeal circuit was recirculated through the cell-saving device (Compact A; Dideco, Mirandola, Italy), filtered and washed, before being transfused to the patient [24]. Anticoagulation was carefully monitored using heparin titration curves (Hepcon, HMS Plus, Medtronic, Minneapolis, MN, USA) with a target activated clotting time (ACT) of 250 s for CABG and 350 s for valvular surgery. The protamine reversal dose was determined after titration by the Hepcon Heparin Management System [25].

Routine postoperative pharmacological management included oral administration of 100 mg acetylsalicylic acid once daily, started on the first postoperative day, and subcutaneous injection of 1 mg/kg of enoxaparin started on the first postoperative day and terminated at hospital discharge. Patients with indwelling drug-eluting coronary stents implanted within 1 year before surgery were supplemented with 75 mg oral clopidogrel daily in addition to acetylsalicylic acid.

### Quantitative variables and statistical methods

Quantitative data are expressed as medians and interquartile ranges (IQR) and compared using the Mann–Whitney *U* test. Qualitative data are described using numbers and percentages and compared using the Fisher’s exact test.

For the primary endpoint, patients were separated into two groups, according to the occurrence of postoperative organ injury (i.e., a SOFA score > 3 at 48 h post-surgery) or not. Their microcirculatory flows measured during iontophoresis were compared according to the three parameters (the peak amplitude, the time to the peak and the AUC at 3 min) using the Mann–Whitney *U* test.

In order to find the best threshold that could allow iontophoresis to predict postoperative organ injury, we built receiver operating characteristics (ROC) curves which explained the occurrence of postoperative organ injury for each significant parameter. A cutoff value was chosen from the point in the ROC curve that was the closest to the top left corner of the graph (i.e., best compromise between specificity and sensitivity).

The ability of ACh iontophoresis, with the chosen cutoff value, to predict the secondary endpoints was searched. Correlation between parameters of interest and quantitative secondary endpoints were tested using the Pearson’s correlation coefficient. In order to confirm that the association between SOFA and the result of the microcirculatory reactivity test remained constant, correlation between significant parameters of microcirculatory reactivity and SOFA at 24, 48 and 72-h was tested using a mixed linear regression model.

In order to investigate the usefulness of microcirculatory measurements in predicting the occurrence of the primary outcome, a multivariable model was constructed. The multivariable model assessing the occurrence of postoperative organ injury by significant parameters of iontophoresis and potentially preoperative confounding variables that significantly differed between patients with and without an event (unadjusted *P* < 0.05). We used the best compromise between specificity and sensitivity on the ROC curve to dichotomize the continuous significant variables. The absence of collinearity between variables was checked on the correlation matrix (*r* < 0.400). Then, each selected variables were entered in logistic regression model. We used the method of backward conditional elimination (cut-off for variable suppression *P* > 0.10). Variables were retained in the model if their suppression resulted in a significant change to the model (*P* < 0.05).

AUC of ROC curve (AUROC) which explained the occurrence of postoperative organ injury by EuroSCORE II, validated commonly used risk score in cardiac surgery in Europe, to predict the primary endpoint was calculated as reference [17]. The accuracy of the probability of occurrence of the primary endpoint according to our model was compared to EuroSCORE II.

A *P*-value < 0.05 was considered to indicate statistical significance. Statistical analyses were performed using SPSS (IBM, Chicago, IL, USA). We did not use an imputation method for missing data. For patients who died in hospital, the worst value of 28 days was assigned for ICU and hospital length of stay. We proceeded in the same way for catecholamine duration if the patient died before the catecholamine weaning.

## Results

### Participants

**Fig. 2:**
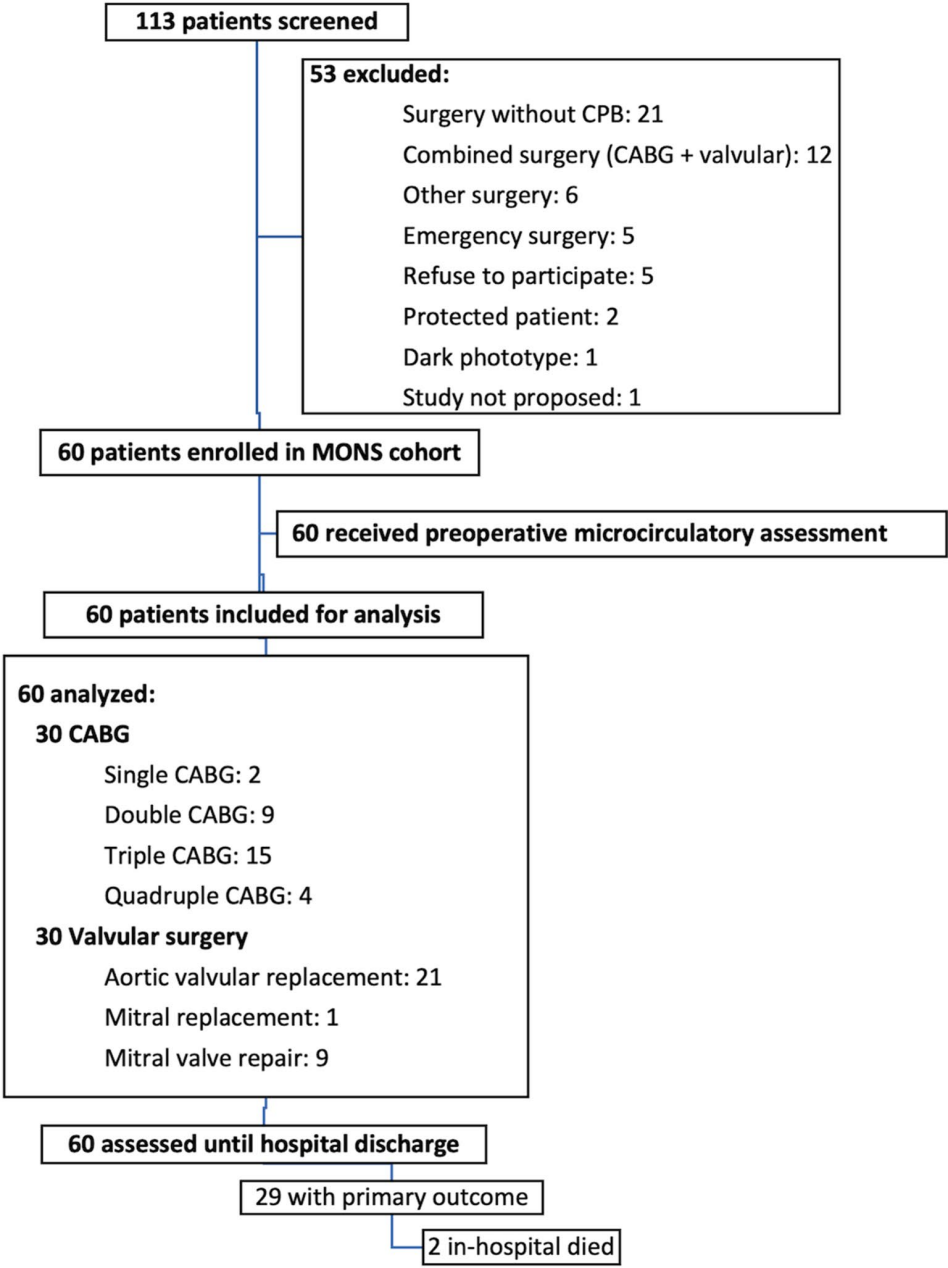
Study flowchart. *CPB* cardiopulmonary bypass, *CABG* coronary arterial bypass grafting

Sixty patients were enrolled in the cohort MONS during the study period. Among them, 30 patients were scheduled to undergo valvular surgery and 30 patients to undergo CABG surgery. Patients were predominantly male (93.3%). The median age was 67 [57–73] years. The patient flowchart is presented in Fig. 2. Patient characteristics are presented in Table 1. The median preoperative hemoglobinemia was 14.4 [13.5–15.3] g/dL. No patient was transfused before surgery. The median duration of surgery was 197 [168–249] min with 95 [72–119] min using CPB and 69 [51–95] min of aortic clamping. All patients were admitted to ICU postoperatively. The median length of ICU stay and hospital stay were 4.7 [4.0–6.1] days and 8.0 [7.0–10.0] days, respectively.

**Table 1:** Characteristics of patients

Characteristic	Total cohort	SOFA > 3 at 48 h	SOFA ≤ 3 at 48 h	*P* value
*N*	60 (100.0)	29 (48.3)	31 (51.7)	–
Male sex	56 (93.3)	27 (45.0)	29 (48.3)	1.000
Age at the enrollment, years	67 [57–73]	70 [66–74]	60 [54–69]	*0.002*
Body mass index, kg/m^2^	27.0 [24.3–30.8]	27.7 [24.3–31.2]	26.8 [24.3–30.5]	0.641
Smokers	30 (50.0)	15 (25.0)	15 (25.0)	1.000
Medical conditions				
Diabetes mellitus	17 (28.3)	6 (10.0)	11 (18.3)	0.258
Hypertension	31 (51.7)	16 (26.7)	15 (25.0)	0.617
Dyslipidemia	25 (41.7)	12 (20.0)	13 (21.7)	1.000
Angina	24 (40.0)	7 (11.7)	17 (28.3)	*0.019*
Myocardial infarction	18 (30.0)	7 (11.7	11 (18.3)	0.405
Peripheral artery disease	5 (8.3)	3 (5.0)	2 (3.3)	0.666
Left ventricle ejection fraction, %	60 [55–65] (*n* = 49)	65 [60–70] (*n* = 26)	60 [54–65] (*n* = 23)	*0.023*
Preoperative atrial fibrillation;	10 (16.6)	9 (15.0)	1 (1.7)	*0.005*
Paroxysmal	5 (8.3)	4 (6.7)	1 (1.7)	
Permanent	5 (8.3)	5 (8.3)	0	
Preoperative biology				
Cockcroft creatinine clearance, mL/min	109 [85–134]	87 [81–113]	124 [107–157]	*< 0.001*
Preoperative platelet count, G/L	218 [195–275]	212 [184–255]	229 [198–284]	0.143
Preoperative hemoglobinemia, g/dL	14.4 [13.5–15.3]	14.6 [13.6–15.5]	14.3 [13.4–15.0]	0.139
Preoperative CRP rate, mg/L^a^	4 [4–4]	4 [4–4]	4 [4–4]	0.282
Preoperative medications				
Beta-blocker	41 (68.3)	21 (35.0)	20 (33.3)	0.585
ACE inhibitors or ARB	38 (63.3)	17 (28.3)	21 (35.0)	0.593
Antiplatelet therapy	41 (68.3)	17 (28.3)	24 (40.0)	0.166
Aspirin therapy	40 (66.7)	16 (26.7)	24 (40.0)	0.100
Dual antiplatelet therapy	21 (35.0)	9 (15.0)	12 (20.0)	0.595
Calcium channel blocker	8 (13.3)	5 (8.3)	3 (5.0)	0.465
Anticoagulant	10 (16.7)	9 (15.0)	1 (1.7)	*0.005*
Statins therapy	40 (66.7)	19 (31.7)	21 (35.0)	1.000
Skin perfusion confounders				
Skin temperature during evaluation, °C	32.9 [32.1–33.7]	32.9 [32.1–33.8]	32.9 [32.0–33.5]	0.412
Elective CABG	30 (50.0)	11 (18.3)	19 (31.7)	0.120
Elective valvular surgery	30 (50.0)	18 (30.0)	12 (20.0)	
EuroSCORE II	0.81 [0.67–1.19]	1.05 [0.76–1.39]	0.80 [0.56–1.06]	*0.020*
Intraoperative confounders				
Duration of surgery, min	197 [168–249]	205 [166–245]	195 [168–249]	0.690
Duration of aortic clamping, min	69 [51–95]	84 [59–119]	64 [48–80]	*0.044*
Duration of CPB, min	95 [72–119]	102 [80–144]	86 [60–114]	0.067
Intraoperative total dose of heparin, UI	22,250 (16,500–28,875)	24,000 [17500–29000]	21,000 [16000–27000]	0.325
Intraoperative norepinephrine	*n* = 41	*n* = 22	*n* = 19	0.958
Dose by weight by duration of CPB, μg/kg/min	0.154 [0.092–0.247]	0.15 [0.09–0.28]	0.18 [0.07–0.25]	

Data are expressed as median [interquartile range] or number (percentage of the entire cohort)

Italic values indicate a *P* value < 0.05

*ACE inhibitor* angiotensin converting enzyme inhibitor, *ARB* angiotensin II receptor blocker, *CABG* coronary arterial bypass grafting, *CRP* C reactive protein, *CPB* cardiopulmonary bypass, *SOFA* Sequential Organ Failure Assessment

^a^Limit of detection < 4 mg/L

### Primary outcome data

Microcirculatory evaluation was performed preoperatively for all included patients. All perfusion data were retained for analysis and were of suitable quality for signal processing. Results of iontophoresis are reported in Table 2. The rate of organ injury (SOFA score > 3 at 48 h) was 48.3%. Patients who postoperatively developed organ injury had a significantly longer time to reach the peak with preoperative iontophoresis of ACh (133 [104–156] s vs 98 [76–139] s for patients without organ injury; *P* = 0.016). The peak amplitude and AUC within 3 min after iontophoresis of ACh were not significantly different between the two groups. Results of iontophoresis of SNP were not significantly associated with organ injury at 48 h (Table 2). The time to reach the peak with preoperative iontophoresis of SNP tended to be different between patients with and without postoperative organ injury. Times to reach the peak with preoperative iontophoresis of ACh and SNP were correlated (*r* = 0.445; *P* < 0.001).

**Table 2:** Association between iontophoresis results and a SOFA score > 3 at 48 h

Iontophoresis	Parameter	Total cohort	SOFA > 3	SOFA ≤ 3	*P*
Acetylcholine	Peak amplitude, LSPU	33.9 [17.0–48.4]	35.5 [24.0–48.3]	26.2 [13.8–49.2]	0.274
	Time to peak, s	111 [88.5–142.7]	133 [104–156]	98 [76–139]	*0.016*
	Area under the curve within 3 min, PU/s	8980 [6701–11122]	9383 [7525–11102]	8179 [5942–11330]	0.277
Nitroprusside	Peak amplitude, LSPU	34.5 [22.0–49.5]	34.2 [25.0–43.9]	35.4 [16.1–50.2]	0.684
	Time to peak, s	214 [164–245]	225 [182–258]	178 [142–235]	0.054
	Area under the curve within 3 min, PU/s	7922 [6699–9843]	7711 [6363–10172]	8225 [6735–9686]	0.684

Results of iontophoresis are presented with medians [interquartile range]. Presented data have been filtered by subtraction of movement artifact. *P* values were obtained for patients with and without an event, using the Mann–Whitney *U* test

Italic value indicates a *P* value < 0.05

*LSPU* laser speckle perfusion units, *SOFA* Sequential Organ Failure Assessment

Patients with postoperative organ injury were older (70 [66–74] years vs 60 [54–69] years for patients without organ injury; *P* = 0.002), had less angina in their medical history (11.7% vs 28.3% for patients without organ injury; *P* = 0.019), lower preoperative Cockcroft creatinine clearance (87 [81–113] mL/min vs 124 [107–157] mL/min for patients without organ injury; *P* < 0.001) and higher left ventricle ejection fraction (65 [60–70]% vs 60 [54–65]% for patients without organ injury; *P* = 0.023). Patients with atrial fibrillation or anticoagulant treatment had significantly more postoperative organ injury than other patients (15.0% vs 1.7% for patients without organ injury; *P* = 0.005) (Table 1). The median length of ICU stay was 5.2 [4.2–6.3] days in the group with organ injury vs 4.1 [3.1–5.3] days for others (*P* = 0.005). The length of hospital stay was also increased in the group with organ injury vs others (9.0 [7.0–14.0] days vs 7.0 [7.0–8.0] days, respectively, *P* = 0.029). The postoperative serum creatinine peak was higher for patients with organ injury, 107 [78–176] vs 80 [72–90] μmol/L for others (*P* = 0.002). The median postoperative peak of arterial lactate was slightly higher for patients with organ injury (2.1 mmol/L [1.6–3.2]) vs others (1.7 mmol/L [1.5–2.1]) (*P* = 0.033).

### Other analyses

#### Cut-off of microcirculatory measurements

**Fig. 3:**
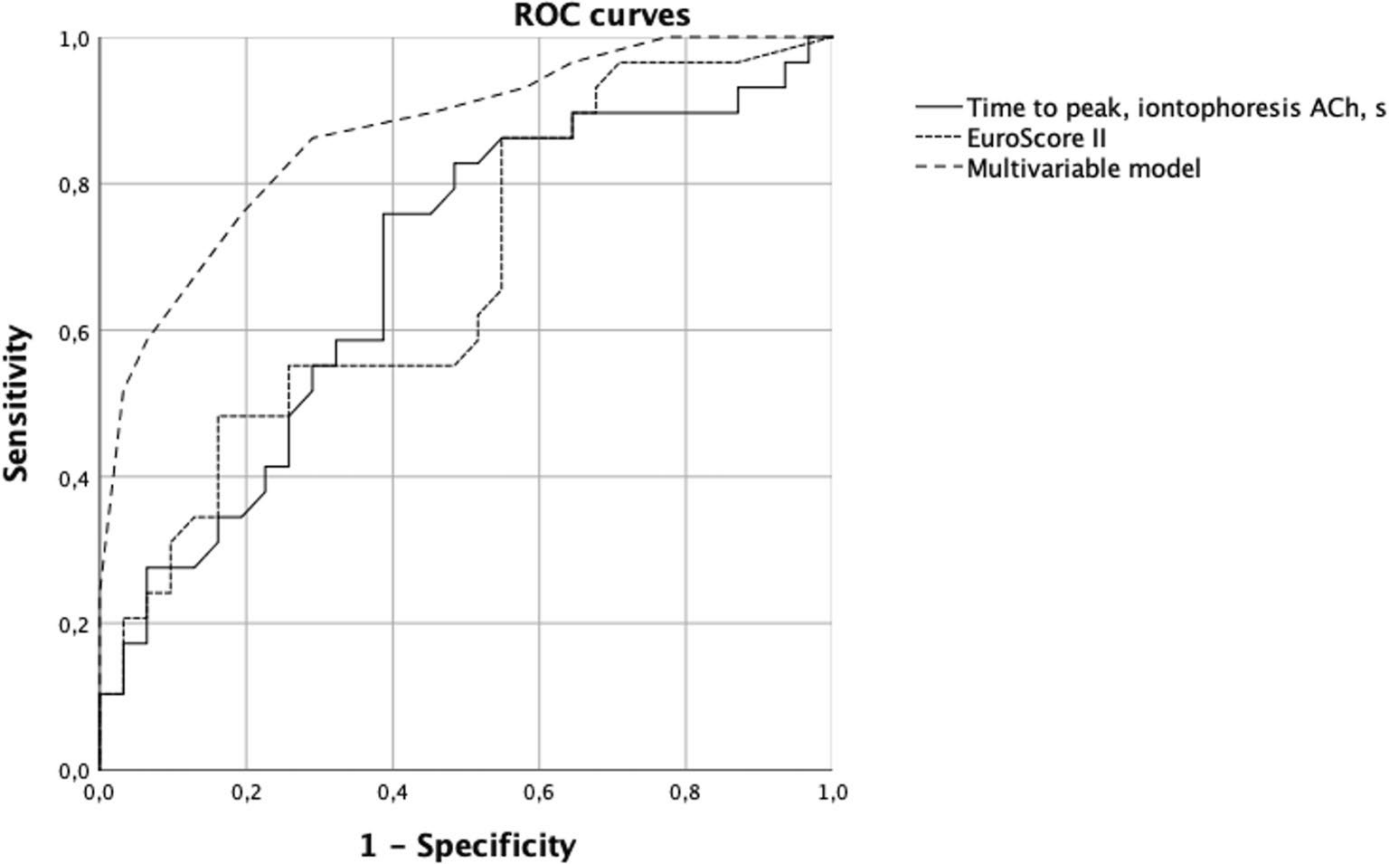
ROC curves for primary judgement criteria

The time to reach the peak at preoperative iontophoresis of ACh had an AUC of ROC curve at 0.681, 95% confidence interval (95% CI 0.545 to 0.818) (Fig. 3). At the threshold of 105 s, the time to reach the peak at preoperative iontophoresis of ACh had 76% (95% CI 56 to 89) and 61% (95% CI 42 to 77) of sensitivity and specificity, respectively, for postoperative organ injury. In the subgroup of patients with time to reach the peak at preoperative iontophoresis of ACh > 105 s, the incidence of organ injury was 76% while it was 24% in subgroup of patients with faster ACh response. In our population, the predictive positive and negative values were 65% (95% CI 46 to 80) and 73% (95% CI 52 to 88), respectively.

A > 105 s time to reach the peak during iontophoresis of ACh was associated with higher age (*P* = 0.006) and lower renal function (*P* = 0.002) (Additional file 1: Table S1). Patients with a prolonged response time had higher EuroSCORE II than other patients (1.09 [0.75–1.35] vs 0.77 [0.56–0.89]; *P* = 0.003). Other patient’s baseline characteristics were not associated with a time to reach the peak during iontophoresis of ACh > 105 s.

#### Secondary endpoints

The correlation between the preoperative time to reach the peak during iontophoresis of ACh and the severity of the organ injury represented by the SOFA score was moderate but significant throughout the postoperative follow-up (at 24 h *r* = 0.426, at 48 h *r* = 0.448, at 72 h *r* = 0.448; *P* < *0.001*). These results were confirmed by the significant correlation between the time to the peak during iontophoresis of ACh and another severity of critical illness score, the SAPS II (*r* = 0.471, *P* < 0.001) (Additional file 1: Figure S1).

A time to reach the peak of microcirculatory flow during iontophoresis of ACh > 105 s was not significantly associated with postoperative significant episode (acute lung injury at 48 h, acute kidney failure of stage > 1, hemodynamic failure at 48 h, late surgical re-intervention and occurrence of episode of atrial fibrillation) (Additional file 1: Table S2). ICU and hospital length of stay (*r* = 0.561, *P* < 0.001; *r* = 0.607, *P* < 0.001, respectively) were moderately correlated with the time to reach the peak of microcirculatory flow during iontophoresis of ACh (Additional file 1: Figure S2). The duration of use of catecholamines (*r* = 0.312, *P* = 0.015) and arterial lactatemia postoperative peak (*r* = 0.379, *P* = 0.003) were also significantly correlated with the time to reach the peak during iontophoresis of ACh with a lower strength of association. No significant correlation was found between the time to the peak during iontophoresis of Ach and the serum creatinine postoperative peak (*r* = 0.207, *P* = 0.113).

#### Multivariable analysis

Preoperative factors associated with organ injury in univariate analysis (Table 1) were included in the logistic regression model. The best compromise between specificity and sensitivity on the ROC curve was used to dichotomize the continuous variables (Additional file 1: Table S3). The time to reach the peak of microcirculatory blood flow during iontophoresis of ACh was the only significant microcirculatory indicator. Preoperative atrial fibrillation (odds ratio [OR] [95% CI] = 24.39 [1.63–364.44]; *P* = 0.021), age greater than 65 years (OR [95% CI] = 10.56 [2.13–52.25]; *P* = 0.004) and the time to reach the peak during iontophoresis of ACh longer than 105 s (OR [95% CI] = 4.81 [1.16–19.94]; *P* = 0.030) were independently associated with postoperative organ injury (Table 3). The history of angina was non-significant adjustment factor retained by the model.

**Table 3:** Multivariable model for predictors of postoperative organ failure

Model	Odds ratio (95% CI)	*P* value
Age > 65 years	10.56 (2.13–52.25)	*0.004*
Preoperative atrial fibrillation	24.39 (1.63–364.44)	*0.021*
Time to reach the peak > 105 s, iontophoresis ACh	4.81 (1.16–19.94)	*0.030*
Preoperative angina	0.28 (0.06–1.22)	0.091

Variables not retained in the model: preoperative anticoagulant therapy (*P* = 0.647), preoperative left ejection fraction > 62% (*P* = 0.268); preoperative creatinine clearance > 105 mL/min (*P* = 0.208)

Odds ratio (95% confidence interval)

Italic value indicate a *P* value < 0.05

*ACh* acetylcholine

#### Comparison with EuroSCORE II

The AUROC of time to reach the peak of microcirculatory flow during iontophoresis of ACh (0.681 [0.545–0.818]) and the AUROC of EuroSCORE II to predict occurrence of organ injury at 48 h were close (0.675 [0.539–0.812]) (Fig. 3). The multivariable model had higher predictive capabilities (0.869 [0.779–0.958]) than EuroSCORE II.

#### Safety and feasibility

No adverse events and no incidents linked to preoperative microcirculatory evaluation were reported.

## Discussion

In this prospective cohort study, preoperative endothelial dysfunction, assessed by iontophoresis of ACh, was associated with postoperative organ injury (i.e., a SOFA score at 48 h > 3) in patients scheduled for cardiac surgery using CPB. Patients with delayed peak of endothelium-dependent vasodilation (> 105 s) had three times more organ dysfunction at 48 h. The time to reach the peak during iontophoresis of SNP was moderately correlated with the time to the peak during iontophoresis of ACh. However, endothelial independent vasodilation, assessed by iontophoresis of SNP was not significantly associated with organ injury at 48 h. This finding suggests that preoperative endothelium-dependent response [15] provides incremental information about the postoperative course and may be related to the pathogenesis of postoperative outcomes. This result was supported by the significant correlation found between the time to reach the peak during iontophoresis of ACh and severity of illness indicators, confirming the clinical impact of organ injury on these indicators.

Several preoperative factors associated with organ injury (i.e., a SOFA score at 48 h > 3) in univariable analysis are well-known predictors of worse outcomes in cardiac surgical population. The actual validated and commonly used risk score in cardiac surgery in Europe, EuroSCORE II, was significantly higher in patients with organ injury and patients with delayed peak at iontophoresis of ACh. In our multivariable model, a longer time to reach the peak during iontophoresis of ACh was an independent factor significantly associated with organ injury (Table 3). After iontophoresis was integrated into the multivariable model, the AUROC was substantially higher than the EuroSCORE II. Thus, the microcirculation evaluation seems to provide additional information compared to the medical conditions or conventional EuroSCORE II risk score. This result supports a role of endothelial function in the risk of postoperative organ injury. Based on this result, a larger study on integration of preoperative microcirculation in a prognostic model could be designed.

Iontophoresis of ACh had been widely used to assess microvascular endothelial-dependent vasodilation [15]. Iontophoresis showed impairment of vasodilation in patients with type 2 diabetes, coronary artery disease and obstructive sleep apnea [26, 27]. These results are concordant with the potential value of using iontophoresis of ACh to identify of a preoperative microcirculatory frailty state in patients with some chronic diseases. In clinical practice, LSCI is recognized as an excellent tool to estimate burn depth and its value in predicting the healing capacity of a burn wound is well established [28]. LSCI is also useful for classification and follow-up of systemic sclerosis [29, 30].

Our findings are supported by several previous studies on peri-operative course. Feng et al. [31] have shown that uncontrolled diabetes results in impaired in vitro arteriolar function before and after on-pump CAGB surgery, alongside enhanced oxidative stress. In patients undergoing vascular surgery, preoperative brachial artery flow-mediated dilation has been shown to be associated with short- and long-term major adverse cardiac events [5, 6, 32]. The brachial artery flow-mediated dilation used by the authors is a non-invasive method for the study of nitric oxide-dependent function. The brachial artery two-dimensional and pulsed Doppler flow velocity signals were obtained above the antecubital crease with a vascular ultrasound system. Hyperemia was observed at arm reperfusion after a brief ischemia. The arm ischemia was induced by inflating a blood pressure cuff on the proximal portion of the arm to occlude the arterial flow (> 200 mmHg) for 5 min. Huang et al*. *[32] showed that preoperative endothelium-dependent flow-mediated dilation and hyperemic flow velocity were lower in patients with an event (cardiac death, myocardial infarction, unstable angina, congestive heart failure or stroke) compared with those without an event (4.4 ± 2.8% vs 7.0 ± 4.9% and 75 ± 39 vs 95 ± 50 cm/s, respectively). However, the brachial artery flow-mediated dilation used by the authors required a period of painful arm ischemia while iontophoresis, used in our study, is totally painless.

The postoperative effect of microcirculation on lactatemia in cardiac surgery using CPB is well known. De Backer et al. [8] found that the severity of postoperative microvascular alterations observed using sublingual video-microscopy is correlated with the lactatemia peak after cardiac surgery. Sublingual video-microscopy has been largely described for monitoring of the microcirculation during cardiac surgery. Microcirculatory impairment assessed by sublingual video-microscopy is associated with inadequate tissue perfusion, leading to peri-operative complications and poor outcomes [33]. Limitations of these methods are linked to oral access, complexity of signal processing (need to be revised manually by an experienced operator), movement and pressure artifacts [15]. In addition, video-microscopy provides only a semi-quantitative measure and no information on the functional capacity of vessels. The better two-dimensional spatial resolution of the LSCI allows for lower spatial variability than the Doppler laser and video-microscopy in vivo do. Although in vivo video-microscopy only allows for morphological analysis, the LSCI allows for the use of a wide variety of microcirculatory reactivity tests [12, 15]. Furthermore, preoperative microcirculation has never been evaluated for the preoperative assessment of patient before a cardiac surgery. In our study, the correlation between postoperative lactatemia was also found. Our method showed the advantage to find this correlation with preoperative measurements and to be easily usable and well tolerated in a conscious patient. In addition, possible movement artifacts are easily subtracted by a non-experimented operator throughout signal processing [22].

One implication of the present study is the possible use of non-invasive examination of endothelial function in the clinical assessment of individual patients. Further work in a larger group of patients is required to build and validate a prognostic model integrating preoperative endothelium-dependent vasodilation. Indeed, currently the large overlap of the time to reach the peak with iontophoresis of ACh between the patients with and without postoperative organ dysfunction did not allow for clinical use of this indicator. For this purpose, one possibility is to define a grey zone around which the lower and upper thresholds could have high sensitivity and specificity values. This method was not applicable in this study because of the low number of patients. Among many outlets, this technique might be used to frame therapeutic decisions about a particular patient’s care. For example, patients with prolonged endothelial response could benefit from less invasive option proposal (percutaneous transluminal coronary angioplasty or transcatheter aortic valve implantation) or specific hemodynamic management such as liberal volume expansion or high-blood pressure target [34, 35]. Conversely, patients with a low risk of organ dysfunction after cardiac surgery could be monitored in an intermediate care unit rather than ICU.

In this study, only preoperative anticoagulant therapy was associated with the occurrence of postoperative organ dysfunction (Table 1). This is a confounding bias. The association was mediated by the preoperative atrial fibrillation (Table 3). A possible protective effect of statin therapy mediated by improvement of the microcirculation was not demonstrated in this study. Preoperative statin therapy was not associated with a significantly different time to reach the peak during iontophoresis of ACh. This result is consistent with previous studies that found neutral or detrimental effects of statins in cardiac surgery particularly about the kidney outcomes [36]. In the same way, despite the detrimental effect of atrial fibrillation, a chronic beta-blocker therapy was not associated with a decrease in organ dysfunction or a shorter time to reach the peak during iontophoresis of ACh [37]. Our study did not identify a drug pathway to correct endothelial-dependent response time, supposing this would be of interest.

Probably the most important limitation of this study (or any such observational study) is that the association does not necessarily indicate causal relationship. Not all of the postoperative outcomes are fully correlated with preoperative microcirculatory flow response. Various other unexpected events as surgical trauma, anatomical or technical difficulties, bleeding, anesthesia stress or hypotension can occur in the peri-operative period for one single type of surgery. For example, depending on the complexity of surgery performed (ranging from single to quadruple bypass), the surgical CABG procedure differs and, as a result, potentially impairs the microcirculation. Koning et al. [9] showed that microcirculation, assessed by sublingual video-microscopy, is preserved during off-pump but not on-pump cardiac surgery. However, each of these factors may have reduced the acuity of the preoperative microcirculatory indicators in our study. Of course, preoperative abnormal microcirculation might be less likely to tolerate occurrence of the peri-operative unexpected events. In this study, the multivariable model was constructed in order to investigate the usefulness of preoperative variables in predicting the occurrence of the primary outcome. For this reason, intraoperative variables were not taking into account in multivariable model construction. This has led to not including the duration of aortic clamping associated with organ injury at 48 h (Table 1) in the model. However, this variable was probably not independent of the complexity of the surgery and comorbidities. Nevertheless, this may have constituted a bias.

The time to endothelial response to ACh was correlated, in our study, with several reference indicators of postoperative outcomes (ICU and hospital length of stay and the duration of use of catecholamine). However, the cut-off that we found at 105 s was not associated with significant postoperative events such as persistent hemodynamic failure. A better evaluation of predictive value of delay of endothelial response for occurrence of each organ failure separately might be achieved.

Limitations of the present study include the small number of subjects and the complexity of our microcirculatory assessment method. The inter- and intra-individual variability of ACh response is approximately 20% [23]. Non-specific effect of the current itself during iontophoresis, named current-induced vasodilation, can interfere with the vasodilation potency of administered drugs [15]. These identified problems may have diminished the discrimination capabilities of iontophoresis. Further studies will be required before this methodology would be considered a clinical tool for preoperative risk assessment. In addition, our data cannot be generalized to women. Finally, we need to investigate which preoperative markers may be the best to identify patients who are at greatest risk of postoperative morbidity and mortality, and hence may most likely require postoperative critical care [38, 39].

## Conclusions

In our study, a longer time to reach the peak of endothelium-dependent vasodilation assessed preoperatively by iontophoresis of acetylcholine was associated with organ injury 48 h after a cardiac surgery using CPB. This well-tolerated and easily feasible technique could be a promising tool for prognostic model of postoperative organ injury after a cardiac surgery.

## Supplementary Information

**Additional file 1: Table S1.** Association between the time to reach the peak during iontophoresis of ACh > 105 s and characteristic of patients. **Figure S1.** Relationship between the preoperative time to reach the peak during iontophoresis of ACh and postoperative severity illness scores. **Table S2.** Association between the time to reach the peak during iontophoresis of ACh > 105 s and qualitative secondary end points. **Figure S2.** Relationship between the time to reach the peak during iontophoresis of ACh and quantitative secondary endpoints. **Table S3.** Dichotomization of continuous variables significantly associated with organ injury.

## Data Availability

The datasets used and/or analyzed during the current study are available from the corresponding author on reasonable request.

## Abbreviations

ACh: Acetylcholine
ACT: Activated clotting time
AUC: Area under the curve
AUROC: Area under the ROC curve
CPB: Cardiopulmonary bypass
CABG: Coronary arterial bypass grafting
ICU: Intensive care unit
IQR: Interquartile ranges
LSCI: Laser speckle contrast imaging
LSPU: Laser speckle perfusion units
ROC: Receiver operating characteristics
SOFA: Sequential Organ Failure Assessment
SNP: Sulfate nitroprusside
