# Supplementary material for: Preoperative endothelial dysfunction in cutaneous microcirculation is associated with postoperative organ injury after cardiac surgery using extracorporeal circulation: a prospective cohort study
Source: Ann Intensive Care. 2021 Jan 7;11:4. doi: 10.1186/s13613-020-00789-y (PMC7790986; doi:10.1186/s13613-020-00789-y)
Supplement: Supplementary file 1 — Additional file 1: Table S1. Association between the time to reach the peak during iontophoresis of ACh > 105 s and characteristic of patients. Figure S1. Relationship between the preoperative time to reach the peak during iontophoresis of ACh and postoperative severity illness scores. Table S2. Association between the time to reach the peak during iontophoresis of ACh > 105 s and qualitative secondary end points. Figure S2. Relationship between the time to reach the peak during iontophoresis of ACh and quantitative secondary endpoints. Table S3. Dichotomization of continuous variables significantly associated with organ injury. [file 13613_2020_789_MOESM1_ESM.docx]

**Additional file 1**

**Table S1 - Association between the time to reach the peak during iontophoresis of ACh >105s and characteristic of patients.**

| Characteristic | Time to peak > 105 s | Time to peak < or = 105 s | *P* value |
| --- | --- | --- | --- |
| N | 34 (56.7) | 26 (43.3) | - |
| Male sex | 31 (51.7) | 25 (41.7) | 0.626 |
| Age at enrollment, years | 70 [61-75] | 62 [53-69] | **0.006** |
| Body mass index, kg/m^2^ | 27.4 [24.8-30.9] | 25.6 [24.1-30.8] | 0.488 |
| Smokers | 20 (33.3) | 10 (16.7) | 0.192 |
| Medical conditions:  Diabetes mellitus  Hypertension  Dyslipidemia  Angina  Myocardial infarction  Peripheral artery disease  Preoperative atrial fibrillation | 11 (18.3)  20 (33.3)  17 (28.3)  12 (20.0)  10 (16.7)  4 (6.7)  7 (11.7) | 6 (10.0)  11 (18.3)  8 (13.3)  12 (20.0)  8 (13.3)  1 (1.7)  3 (5.0) | 0.566  0.297  0.188  0.435  1.000  0.377  0.491 |
| Left ventricle ejection fraction, % | 63 [54-70] (n=31) | 60 [55-65] (n=18) | 0.247 |
| Preoperative biology:  Cockcroft creatinine clearance, ml/min  Preoperative platelet count, G/L  Preoperative hemoglobinemia, g/dL  Preoperative CRP rate, mg/L * | 91 [82-115]  218 [192-283]  14.0 [13.4-15.3]  4 [4-4] | 121 [106-157]  223 [196-266]  14.8 [14.2-15.2]  4 [4-4] | **0.002**  0.835  0.133  0.503 |
| EuroSCORE II | 1.09 [0.75-1.35] | 0.77 [0.56-0.89] | **0.003** |
| Preoperative medications:  Beta-blocker  ACE inhibitors or ARB  Antiplatelet therapy  Aspirin therapy  Dual antiplatelet therapy  Calcium channel blocker  Anticoagulant  Statin therapy | 22 (36.7)  23 (38.3)  23 (38.3)  23 (38.3)  11 (18.3)  7 (11.7)  8 (13.3)  26 (43.3) | 19 (31.7)  15 (25.0)  18 (30.0)  17 (28.3)  10 (16.7)  1 (1.7)  2 (3.3)  14 (23.3) | 0.581  0.589  1.000  1.000  0.785  0.122  0.163  0.097 |
| Skin perfusion confounders:  Skin temperature during evaluation, °C | n = 31  32.9 [32.1-33.7] | n = 24  32.7 [32.1-33.6] | 0.759 |

Data are expressed as median [interquartile range] or number (percentage of the entire cohort).

* Limit of detection < 4 mg/L

ACE inhibitor: Angiotensin converting enzyme inhibitor. ARB: Angiotensin II receptor blocker. CRP: C reactive protein.

**Figure S1 – Relationship between the preoperative time to reach the peak during iontophoresis of ACh and postoperative severity illness scores.**

**
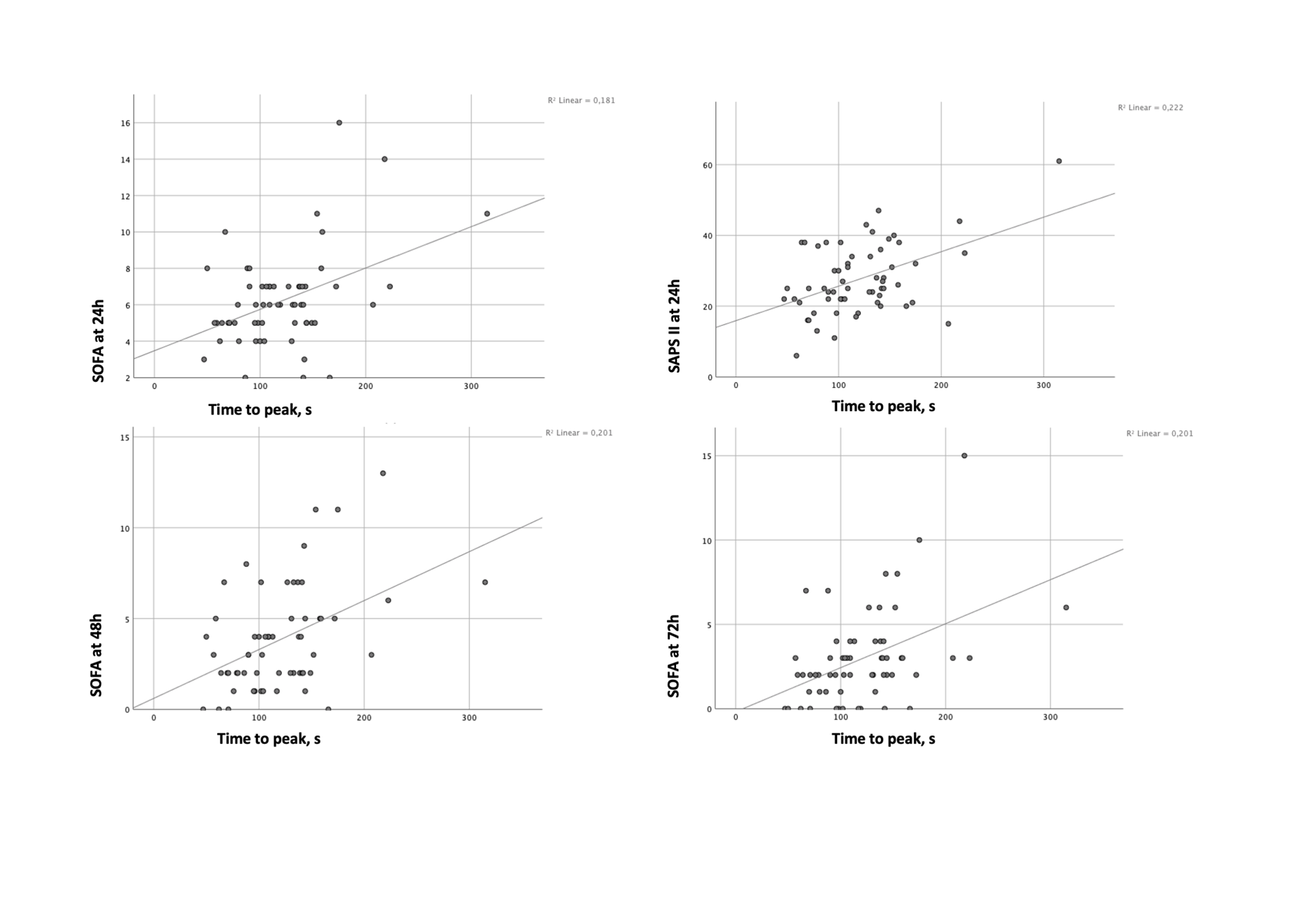
**

X-axis: Time to reach the peak of microcirculatory flow during iontophoresis of acetylcholine, second.

Y-axis: postoperative severity illness scores.

**Table S2 – Association between the time to reach the peak during iontophoresis of ACh >105s and qualitative secondary end points.**

| **Outcomes** |  | **Time to peak, s** | | ***P*** |
| --- | --- | --- | --- | --- |
|  |  | > 105 s | ≤ 105 s |  |
| **Acute lung injury at 48h** | Yes  No | 18  7 | 16  19 | 0.064 |
| **Acute kidney injury > stage I** | Yes  No | 15  19 | 13  13 | 0.795 |
| **Hemodynamic failure at 48h** | Yes  No | 10  24 | 3  23 | 0.122 |
| **Late surgical re-intervention** | Yes  No | 4  30 | 2  24 | 0.689 |
| **Episode of atrial fibrillation** | Yes  No | 17  17 | 12  14 | 0.800 |

*P* value obtained by Fisher’s exact test.

**Figure S2 – Relationship between the time to reach the peak during iontophoresis of ACh and quantitative secondary endpoints.**


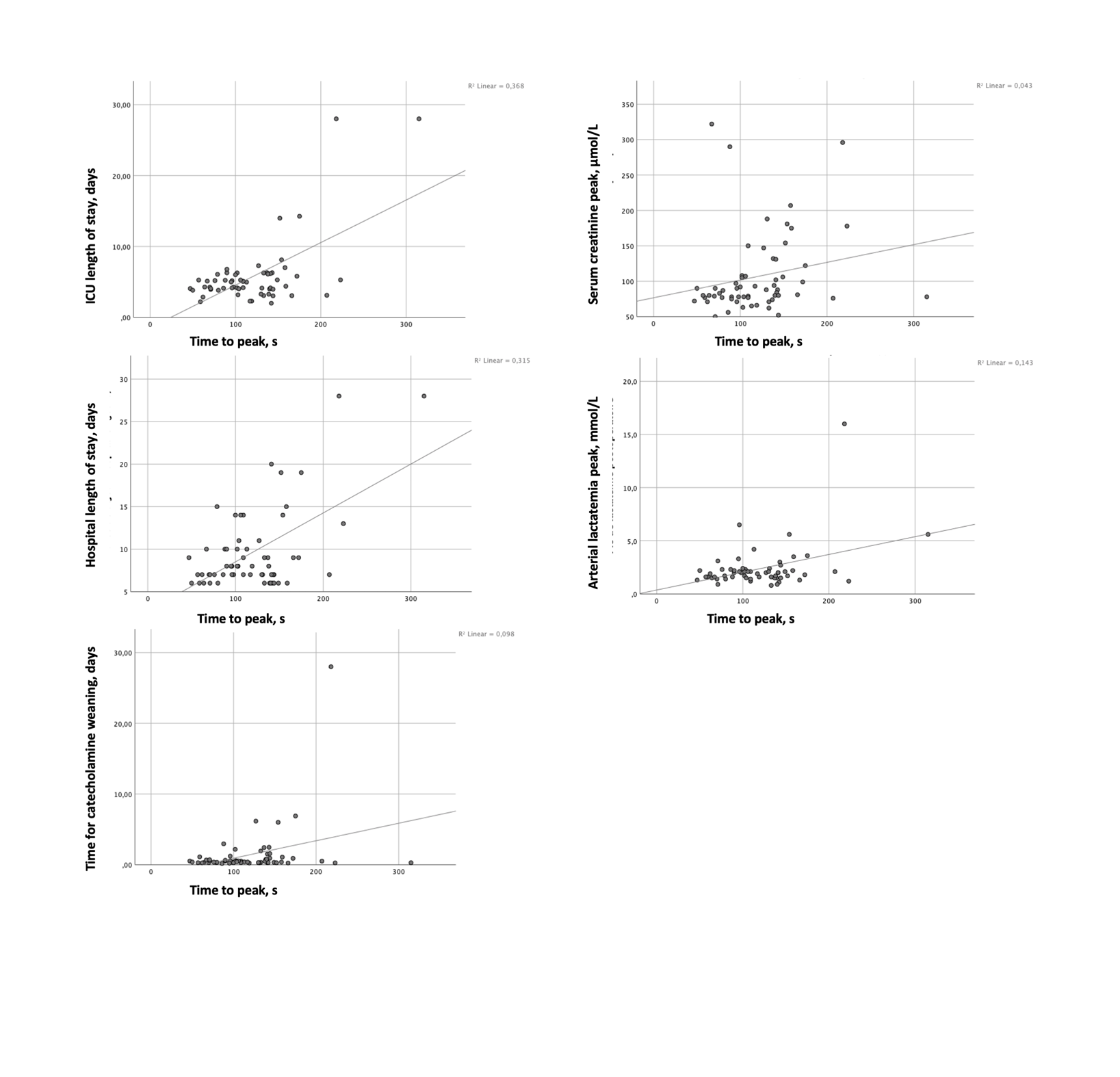


X-axis: Time to reach the peak of microcirculatory flow during iontophoresis of acetylcholine, second.

Y-axis: secondary endpoints.

**Table S3 – Dichotomization of continuous variables significantly associated with organ injury.**

| **Variable** | **Age (n=60)** | **LVEF (n=49)** | **CrCl (n=60)** |
| --- | --- | --- | --- |
| **ROC curves** | 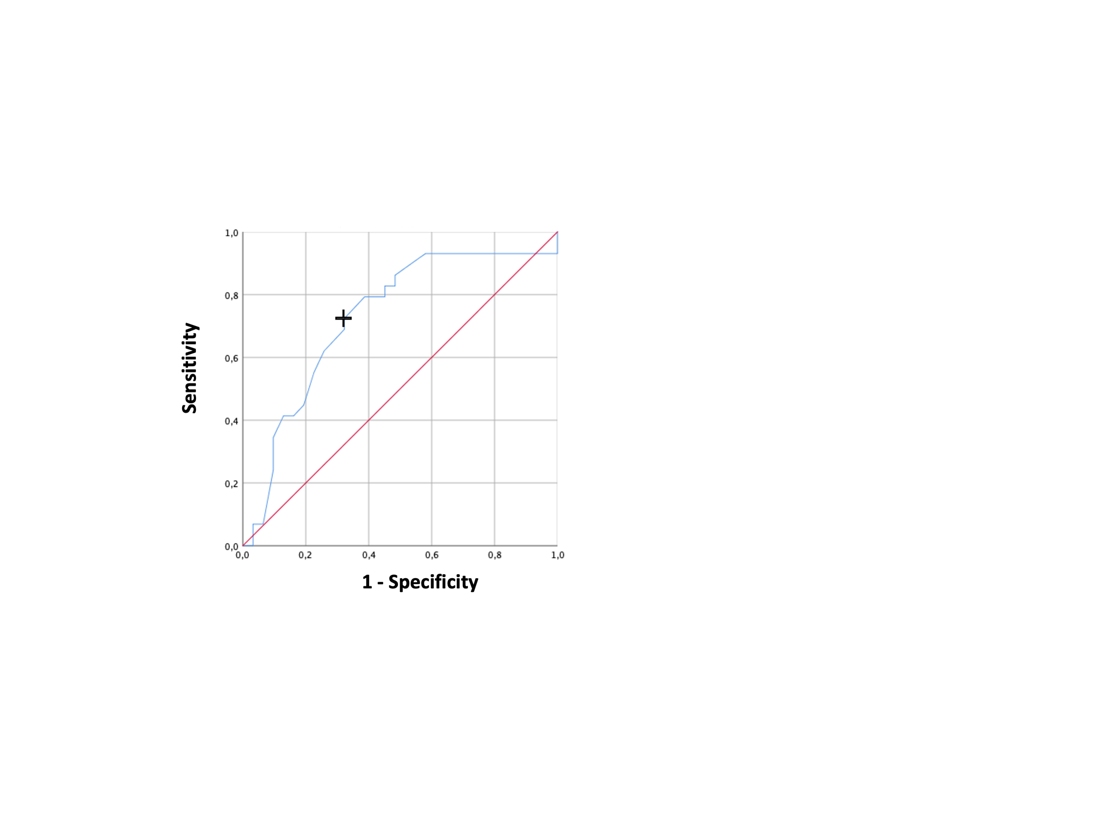 | 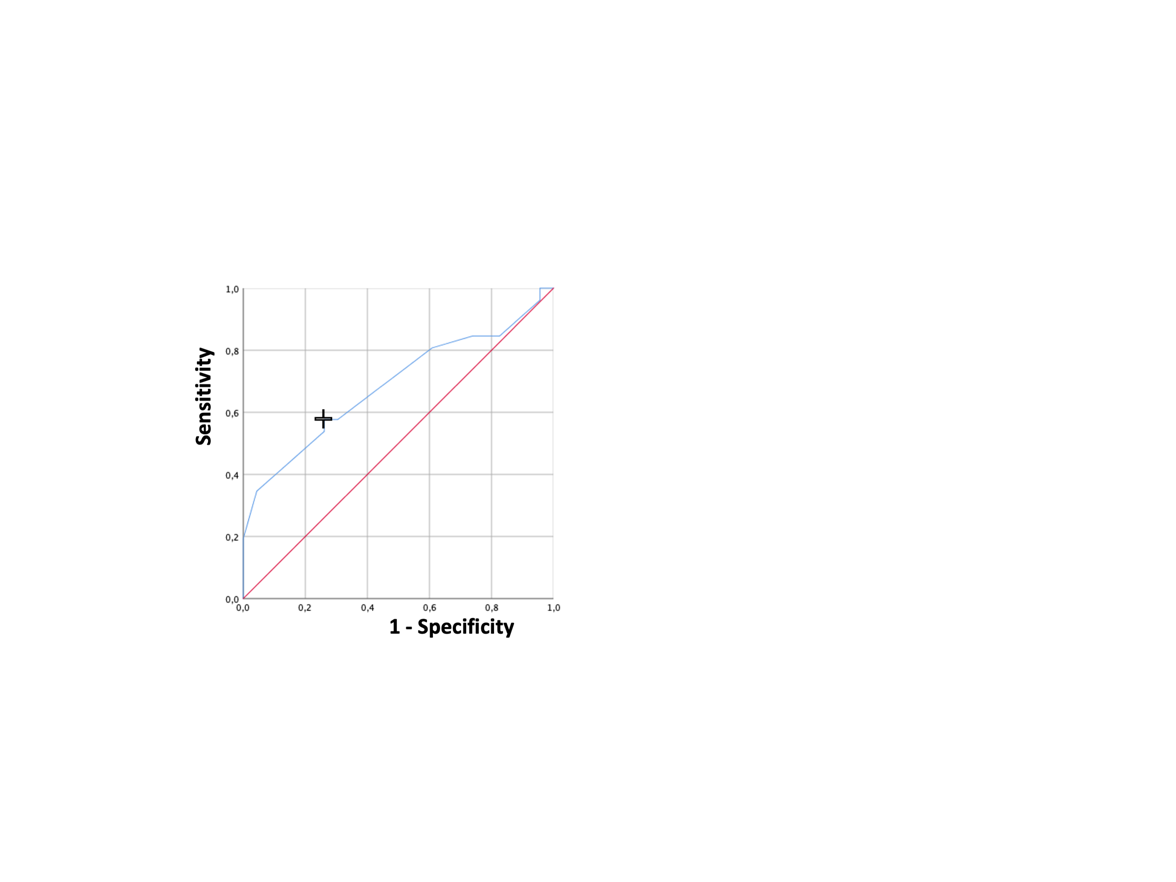 | 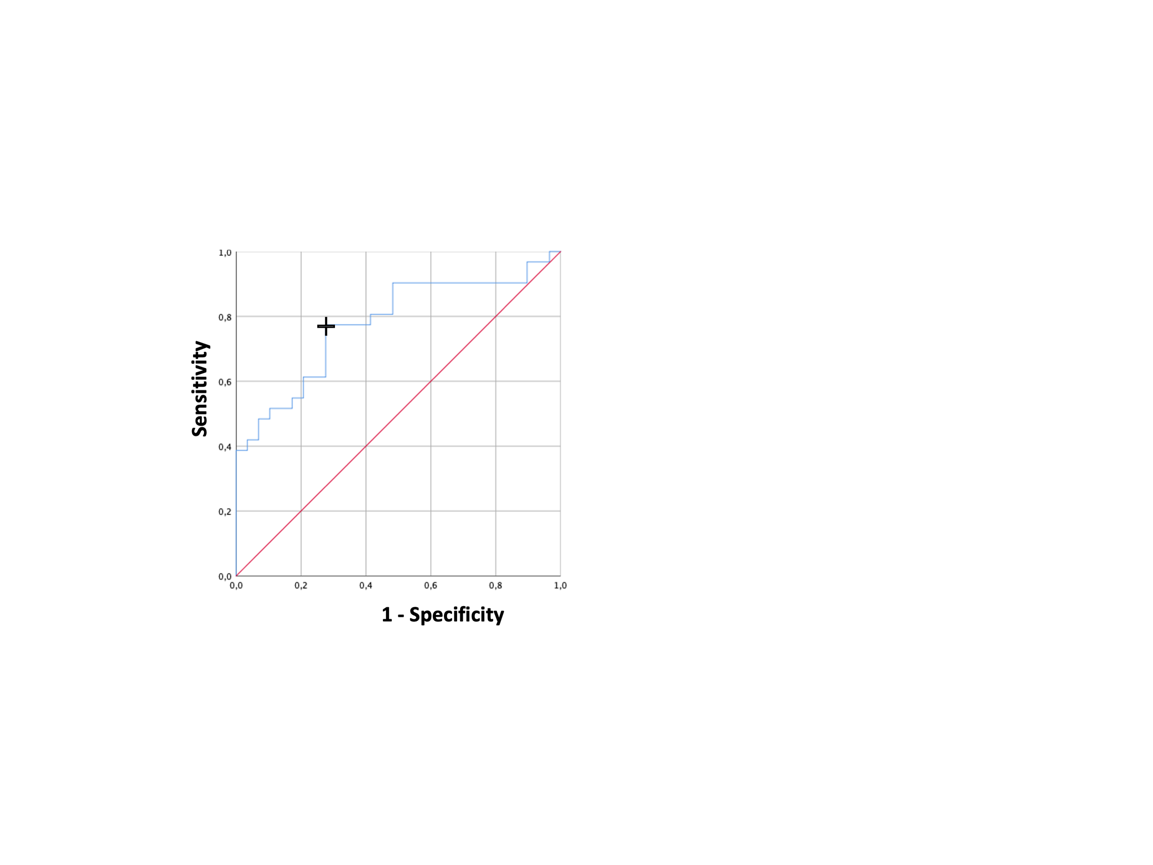 |
| **Cut-off** | 65 years | 62 % | 105 ml/min |
| **Sensitivity, %** | 79 [64-94] | 58 [39-77] | 72 [56-89] |
| **Specificity, %** | 62 [44-78] | 74 [56-92] | 77 [63-92] |

The best compromise between specificity and sensitivity on the ROC curve for primary judgement criteria was used to dichotomize the continuous variables.

LVEF: Left ventricle ejection fraction

CrCl: Cockcroft creatinine clearance
